# The DNA damage response: Balancing the scale between cancer and ageing

**DOI:** 10.18632/aging.100248

**Published:** 2010-12-16

**Authors:** Elena G. Seviour, Shiaw-Yih Lin

**Affiliations:** Department of Systems, Biology, M. D. Anderson Cancer Center, Houston, TX 77054, USA

**Keywords:** aging, cancer, DNA damage, p53, senescence, ATM

## Abstract

Defects in the DNA damage response often lead to an increased susceptibility to cancer, and so the DDR presents an interesting set of novel therapeutic targets. The maintenance of genomic integrity by the DDR has also been found to be involved in the process of organismal ageing. While the removal of cells containing damaged DNA can be beneficial in the prevention of cancer, it may contribute to both normal and pathological ageing.

## INTRODUCTION

Given the frequency at which DNA lesions occur (approximately 10^4^ per cell per day [1]), a complex system of damage detection and repair is required in order to preserve the integrity of the genome. This system is termed the DNA damage response (DDR), and encompasses: the recognition of DNA damage; the transduction of signals through appropriate pathways; and the activation of cellular responses ranging from DNA repair and chromatin remodeling to the activation of cell death if the damage is irreparable [2-4].

DNA lesions can be caused by either endogenous (reactive oxygen species (ROS) resulting from metabolic processes) or exogenous (ionizing radiation (IR), UV) agents. The repair pathway activated in res-ponse to such agents is dependent on the type of lesion generated. Base excision repair (BER) and nucleotide excision repair (NER) pathways are typically activated in response to damage to individual DNA bases [5], while breaks in one (SSBs) or both (DSBs) require repair by mechanisms such as homologous recombination (HR), single strand annealing (SSA) or non-homo-logous end joining (NHEJ). As these processes are reviewed elsewhere [6-8], they will not be covered by this review. Instead, it will focus on the interplay between some of the key components of the signaling pathways preceding DNA repair, the roles of these proteins in the maintenance of genomic stability, and will finally seek to address the role of the DDR in both cancer and ageing.

### The DDR: functions of and interplay between the key players

The DDR comprises multiple proteins, and a complicated network of signaling pathways to ensure that the processes of DNA repair, cell cycle arrest or the triggering of the apoptotic cascade are correctly regulated. Since the individual elements of the DDR have been reviewed at length elsewhere, this paper will aim to give a brief overview of the key components in order to further discuss how the DDR can be targeted to treat cancer.

#### ATM.

ATM (ataxia telangiectasia mutated) is a member of the phosphatidylinositol 3-kinase related family of serine/threonine protein kinases (PIKKs) [9]. ATM is critical in the immediate response of cells to DSBs and the subsequent switch to ATR activation following DNA end resection [10]. Mutation of ATM in humans leads to the condition ataxia telangiectasia (A-T), which is characterized by: progressive neurodegeneration; immunodeficiency; genomic instability; clinical radio-sensitivity; and a predisposition to cancer, in particular lymphomas as a result of inappropriate signaling following programmed DSBs during V(D)J recombination in T-cells [11,12]. The recruitment of ATM following DSBs is mediated by the Mre11/Rad50/NBS1 (MRN) complex [13] in response to chromatin decondensation and relaxation of the double helix torsional stress [14]. Acetylation of ATM by the histone acetylase Tip60 stimulates ATM autophosphorylation, resulting in the dissociation of inactive homodimers into monomers, and the phosphorylation of downstream substrates [15].

Multiple proteins have been shown to be phosphorylated downstream of ATM, including the known tumor suppressor protein p53, structural maintenance of chromosomes (SMC) 1, which is known to engage the S phase checkpoint, the breast and ovarian cancer susceptibility protein BRCA1 and the checkpoint kinase Chk2 [16-19]. These will be discussed in more detail later.

#### ATR.

ATR (ATM-Rad3-related) is also a member of the PIKK family, and while being related to ATM, plays a distinct role in the DDR. Loss of ATR has been shown in mouse models to convey embryonic lethality [20], suggesting a critical role for the protein in development. Humans surviving with ATR mutations display a condition called Seckel syndrome, the phenol-type of which includes growth retardation and micro-cephaly [21].

The initial step in ATR signaling is the binding of replication protein A (RPA) to single stranded DNA (ssDNA), which recruits the ATR-ATRIP complex to the DNA damage. The recognition of neighboring DNA ends by the RAD9-RAD1-HUS1 (9-1-1) complex brings the protein TOPBP1 into the vicinity of ATR-ATRIP to stimulate ATR activation [22-24]. As with ATM, this activation results in the phosphorylation of multiple substrates, such as Chk1 [25]. There is some overlap between the ATM and ATR pathways at the substrate level, with both having been shown to phos-phorylate various substrates, including p53 and BRCA1 [26-28].

#### BRCA1.

Following DNA strand breaks, BRCA1 has been found to localize to IR-induced nuclear foci. This localization has been shown to regulate the activation state of many proteins downstream of both ATM and ATR, thereby suggesting a possible means of cross-talk and overlap between the two pathways [29]. BRCA1 has also been found to form distinct macromolecular complexes, which allow BRCA1 to participate in multiple DDR-related functions, including regulation of the G_1_/S and G_2_/M checkpoints, and the repair of DSBs by HR (reviewed in [30]).

The induction of G_1_/S arrest by BRCA1 is known to require several other proteins. The inhibition of gene transcription is regulated by the interaction of hyposphosphorylated pRB with the E2F transcription factor. This hyposphosphorylated state of pRB is thought to be maintained by its interaction with BRCA1 [31]. Cell cycle arrest may also be a result of the regulation of p53 phosphorylation by the BRCA1-BARD1 complex, as the phosphorylation of p53 is critical for its activation [32].

The intra-S phase checkpoint is also regulated by BRCA1, through the BRCA1B complex containing BRCA1, BACH1 and TOPBP1 (reviewed in [30]). This complex mediates the firing of replication origins by regulating the loading of the licensing factor CDC45L onto the DNA [33]. The BACH1-TOPBP1 interaction has also been shown to be essential for the optimal loading of RPA onto chromatin, suggesting that BRCA1 is critical in the maintenance of DNA replication through regulating the response of cells to stalled replication forks [34].

The BRCA1C complex, comprising BRCA1, the carboxy-terminal binding protein interacting protein (CtIP) and the MRN complex, has an essential function in regulating the G_2_/M checkpoint. The interaction of CtIP and MRN has been found to regulate the resection of DNA ends [35], a process also mediated by BRCA1 [36,37], thereby regulating the function of the ATR-ATRIP complex at regions of ssDNA, and so regulating the progression of the cell through the G_2_/M checkpoint.

#### BRIT1.

Another protein recently shown to interact with multiple members of the DDR is the repressor of hTERT, BRIT1. This protein functions at multiple stag-es in the DDR, including the regulation of the G_2_/M checkpoint, stimulation of BRCA1 and Chk1 expression and the interaction and recruitment of the BRCA2/ RAD51 complex for the execution of HR. The documented failure of various DDR proteins including NBS1, phosphorylated ATM and ATR to localize to sites of DNA damage in the absence of BRIT1 suggests that BRIT1 functions upstream of these proteins as a scaffold to bridge phosphorylated H2AX and the DDR (reviewed in [38]).

In 2009, BRIT1 was shown to recruit the SWI-SNF chromatin remodeling complex to sites of DNA damage in an ATM/ATR-dependent manner. This recruitment is thought to facilitate the unwinding of chromatin following DNA damage, thereby allowing access of the required repair proteins. This shows that BRIT1, therefore, plays a critical role in both HR and NHEJ mediated repair [39].

**Figure 1.**
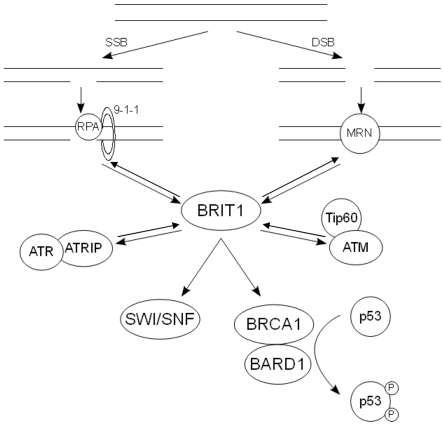
The action of the key DDR components following DNA damage. Following SSBs, RPA is recruit-ed to the ssDNA along with the 9-1-1 complex. This in turn recruits the ATR-ATRIP complex, allowing ATR to phosphorylate and activate its downstream substrates. Damage that results in DSBs causes the recruitment of the MRN complex, which binds and activates ATM. The pathways at least partially con-verge on BRIT1, which regulates the expression of BRCA1. The BRCA1-BARD1 complex in turn regulates the phosphorylation state of p53.

The interplay between BRIT1, BRCA1 and the other upstream DDR components discussed here is illustrated in figure 1.

#### Checkpoint kinases.

The checkpoint kinases Chk1 and Chk2 are widely considered to be the major effectors of the DDR in regulating cell cycle checkpoints and coordinating this with DNA repair.

**Figure 2.**
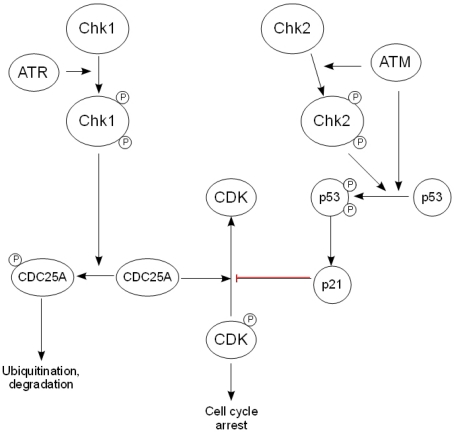
The downstream effectors of the DDR. After activation by ATR, Chk1 phosphorylates the CDC25 family of phosphatases, thereby targeting them for ubiquityn-ation and subsequent degradation and preventing the activation of cyclin-dependent kinases. Chk2 is activated by ATM and phosphorylates p53, causing its stabilization and activation, while ATM also activates p53 directly. This in turn regulates the expression of the CDK inhibitor p21, leading to arrest of the cell cycle.

As with ATR, Chk1 has found to be critical for embryonic development, suggesting that it is active at multiple stages within the cell cycle [40]. In response to DNA damage and ATR activation, the adaptor molecule claspin is phosphorylated, which results in the recruitment of Chk1 to ATR. Following its phosphorylation, Chk1 is released from the chromatin in its active form, allowing it to phosphorylate the CDC25 family of phosphatases [41]. This targets the phosphatase for degradation via the ubiquitin/proteasome pathway, and so prevents it from dephosphorylating and activating CDKs, leading to cell cycle arrest at either the G_1_/S, intra-S phase or G_2_/M checkpoint [42] (Fig. 2).

In contrast, Chk2 is not considered to be vital for embryonic development, but is believed to be activated following DNA damage. Although Chk2 has been found to phosphorylate CDC25A, its role in the DDR is thought to be related more to its effect on p53 stabilization than cell cycle arrest [43].

#### p53.

The frequency of p53 mutation and loss in human cancers suggests a critical role for p53 in the maintenance of genomic integrity. p53 can be phosphorylated by a number of DDR proteins, including ATM, ATR and Chk2 [17,44,45]. This phosphorylation reduces the binding of p53 to MDM2, resulting in stabilization and activation of the p53 protein.

One of the key activities of p53 is thought to be the induction of expression of p21, a known regulator of both the G_1_/S and G_2_/M transitions through its inhibition of cyclin-dependent kinases (CDK) [46]. A further role for p53 in tumor suppression is the regulation of the apoptotic cell death pathway. However, this falls outside the scope of this review and so will not be discussed here. Figure 2 shows an overview of the pathways connecting the checkpoint kinases, p53 and the mechanism of regulation of cell cycle arrest.

### The DDR, genomic stability and cancer

Over 30 years ago it was established that cells within a tumor are derived from a single genetically unstable cell, and that the population as a whole continues to acquire further chromosomal abnormalities over time [47]. However, the precise mechanisms of acquisition of these abnormalities have remained unclear. Hereditary cancers are often characterized by the presence of a specific type of genomic instability, termed chromosomal instability (CIN). In these cancers, CIN can often be attributed to mutation in DNA repair genes, suggesting that the drive behind tumor development is the increase in spontaneous genetic mutation resulting from a lack of appropriate management of DNA damage [48]. A second form of genomic instability, termed microsatellite instability (MIN), is also associated with defects in DNA repair, namely the mismatch repair system [49]. However, in non-hereditary sporadic tumors, the picture is less clear.

Many of the DDR components including BRCA1 [50] and BRIT1 [38] are known to be lost or mutated in human tumors, and patients suffering from ataxia telangiectasia are known to be susceptible to tumors, as mentioned above.

While the loss of BRCA1 has been shown to lead to the development of mammary tumors in mouse models, the genetic diversity within those tumors suggests that the loss of BRCA1 is not directly responsible for tumorigenesis. It is more likely, therefore, that the role of BRCA1 in the initiation of cancer is a result of its effects on DNA repair and the maintenance of genomic integrity [51]. These mouse models, coupled with the study of human BRCA1 −/− tumors, has revealed a prevalence for p53 mutations in these tumors, which is likely to be caused by the decrease in genomic stability associated with the defects in DNA repair (reviewed in [52]).

Similar to BRCA1 −/− tumors, BRIT1 −/− tumors also display numerous chromosomal aberrations. Interest-ingly, BRIT1 is expressed on the short arm of chromosome 8, a region which has been found to be altered in various forms of cancer [53-56]. A significant increase in cancer susceptibility was also noted in mice crossed from both BRIT1 −/− and p53 −/− backgrounds [38]. Taken together, these data suggest that the loss of cell cycle checkpoints confers a selection advantage to cells with DNA repair defects, thereby triggering tumorigenesis in genetically unstable cells. The reduction in BRIT1 expression correlates significantly with an increase in genomic instability, as well as with the metastatic potential of the tumor. Further studies will be required to determine whether this involvement in metastasis is a result of acquired genetic mutations resulting from DNA repair defects, or whether other binding partners of BRIT1 are required for this process.

### The DDR and ageing

In addition to its role in the maintenance of genomic integrity, the DDR has been hypothesized to play a critical role in organismal ageing. Ageing, resulting from the accumulation of damage to molecules, cells, organs and tissues over time, is believed to be caused by two cellular processes: senescence and apoptosis.

#### Senescence.

Senescence was first described by Hayflick and Moorhead in 1961 when it was noted that fibroblasts entered a state of permanent growth arrest following serial cultivation; a fate that was not shared by cancer cells [57]. Further study has revealed different forms of senescence, namely replicative senescence and oncogene-induced senescence, both of which involve aspects of the DDR.

Replicative senescence results from the progressive shortening of telomeres with repeated rounds of cell replication. In 2007, Feldser and Greider demonstrated that the shortening of telomeres in mice was related to a suppression of tumor incidence as a result of induction of a p53-dependent senescence pathway. This p53 response to critically shortened telomeres has been demonstrated to be a result of activation of the DNA damage response (reviewed in [58]), since the shortening of telomeres results in their uncapping and their recognition as damaged DNA [59].

**Figure 3.**
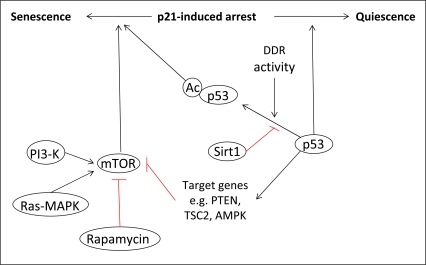
The role of p53 in senescence. The determination of cell fate following p21-induced cell cycle arrest is dependent on the activity of p53 towards mTOR. Under conditions where p53 can inhibit mTOR via the transcriptional activation of specific target genes, cells will enter quiescence. However, when p53 cannot inhibit mTOR, cells will become senescent. The transcriptional activity of p53, and so its activity towards mTOR, can also be regulated by post-translational modifications such as acetylation, which is linked to the activity of the DDR.

However, the role of p53 in the induction of a senescent phenotype has been debated by recent studies, which have shown that p53 can either activate or suppress senescence. The determination of cell fate has been postulated to depend on the specific transcriptional activities of p53. Several recent reports have proposed a mechanism by which p21-induced cell cycle arrest could be converted into either a quiescent or senescent phenotype, depending on the activity of p53 and its inhibition of the protein kinase mammalian target of rapamycin (mTOR) [60-62]. Briefly, it has been suggested that under conditions where p53 is able to inhibit the mTOR pathway, cells will become quiescent. However, when p53 is unable to inhibit mTOR signaling, the drive will be towards senescence. A further level of complexity to the role of p53 in cell fate determination was also suggested by Vigneron *et al.*[63]. This publication highlighted the importance of the histone deacetylase Sirt1 in the regulation of p53 transcriptional activity, suggesting that the induction of senescence by chronic DDR signaling may be linked to decreased levels of Sirt, and so increased levels of p53 acetylation. The results of these studies are summarized in figure 3.

p53 is not the only DNA damage response protein to be associated with replicative senescence. Studies in mice have shown that the deletion of ATM causes an increase in both chromosomal end-to-end fusion events and cell cycle-dependent telomere loss. These events were accompanied by a premature ageing phenotype, with symptoms including increased hair graying, alopecia and marked weight loss [64]. A further study also demonstrated that mice expressing a mutant form of BRCA1 also show premature ageing, accompanied by an increase in cellular senescence [65]. The ageing phenotype in this model was also accompanied by an increased susceptibility to certain cancers. While this might seem contradictory to the enhanced senescence in these mice, senescent cells have been noted to modify the tissue microenvironment by the secretion of degradative enzymes, cytokines and growth factors. This is thought to synergize with the accumulation of DNA damage over time to encourage cancer growth [66].

Senescence can also be induced by the overexpression of oncogenes. This has recently been reviewed elsewhere [67] and so will not be discussed in detail. Briefly, the expression of oncogenes is thought to induce senescence by multiple means, including the induction of DNA damage resulting from both the generation of reactive oxygen species (ROS) and the hyper-replication of DNA. Both of these mechanisms activate the DDR, which induces senescence as described for replicative senescence above.

#### Apoptosis.

The accumulation of DNA damage does not necessarily lead to cellular senescence. The activation of p53 by DNA damage has been well documented, and its role in the regulation of expression of pro-apoptotic proteins has been recently reviewed [68]. The fact that the majority of tumors lose the expression of functional p53 underlines its importance as a regulator of cell death processes. In the context of ageing, the apoptotic function associated with p53 activation has been previously documented in terms of the decline of the immune system associated with an increase in apoptosis [69-71]. A mouse model in which p53 is constitutively activated also showed that, while high levels of p53 protect against cancer, it also accelerates the ageing process by reducing the mass of various tissues [72]. The human condition ataxia-telangiectasia, which results from mutations in ATM, is associated with substantial neurodegeneration. This has been shown in a mouse model to result from an accumulation of neurons harboring genomic damage, due to the inability of the mutant ATM protein to stimulate the p53 apoptotic cascade [73]. Chk2 has also been shown to regulate apoptosis in a p53-dependent manner *in vitro*[74] and *in vivo*[75] in response to DNA damage.

## CONCLUSION

The DDR is a complex network of proteins, comprising DNA damage recognition, signal transduction, transcriptional regulation, cell cycle control and DNA repair. The maintenance of the DDR is essential for faithful replication of the genome, and so is critical for cellular survival. The loss of certain DDR components can lead to an increased susceptibility to cancer due to the ensuing genomic instability and the subsequent mutation to genes required for cellular replication and division. The DDR is also involved in the induction of senescence and apoptosis when the damage cannot be repaired. While this can prolong longevity during early stages of life due to the suppression of tumorigenesis, it may become detrimental in ageing due to the loss of stem and progenitor cells for renewal. This is a phenomenon referred to as antagonistic pleiotropy, and it highlights the importance of carefully balanced cell signaling cascades and regulatory systems in the maintenance of survival. Further studies of the roles of DDR-associated proteins, along with the discovery of new ones, will therefore not only enhance our understanding of cancer and mechanisms to treat it, but will also enhance our understanding of the ageing process. This may uncover ways to treat premature ageing or other age-related pathologies, such as the decline of the immune system in the elderly.
